# The Function of Immunoproteasomes—An Immunologists’ Perspective

**DOI:** 10.3390/cells10123360

**Published:** 2021-11-30

**Authors:** Bart L. van den Eshof, Lobna Medfai, Emanuele Nolfi, Magdalena Wawrzyniuk, Alice J. A. M. Sijts

**Affiliations:** Department of Infectious Diseases and Immunology, Faculty of Veterinary Medicine, Utrecht University, Yalelaan 1, 3584 CL Utrecht, The Netherlands; b.l.vandenEshof@uu.nl (B.L.v.d.E.); l.medfai@uu.nl (L.M.); e.nolfi@uu.nl (E.N.); m.wawrzyniuk@uu.nl (M.W.)

**Keywords:** immunoproteasome, antigen processing, CD8 T cell responses, immune protection

## Abstract

Proteasomes are responsible for intracellular proteolysis and play an important role in cellular protein homeostasis. Cells of the immune system assemble a specialized form of proteasomes, known as immunoproteasomes, in which the constitutive catalytic sites are replaced for cytokine-inducible homologues. While immunoproteasomes may fulfill all standard proteasome’ functions, they seem specially adapted for a role in MHC class I antigen processing and CD8^+^ T-cell activation. In this way, they may contribute to CD8^+^ T-cell-mediated control of intracellular infections, but also to the immunopathogenesis of autoimmune diseases. Starting at the discovery of its catalytic subunits in the genome, here, we review the observations shaping our current understanding of immunoproteasome function, and the consequential novel opportunities for immune intervention.

## 1. Introduction

**Figure 1:**
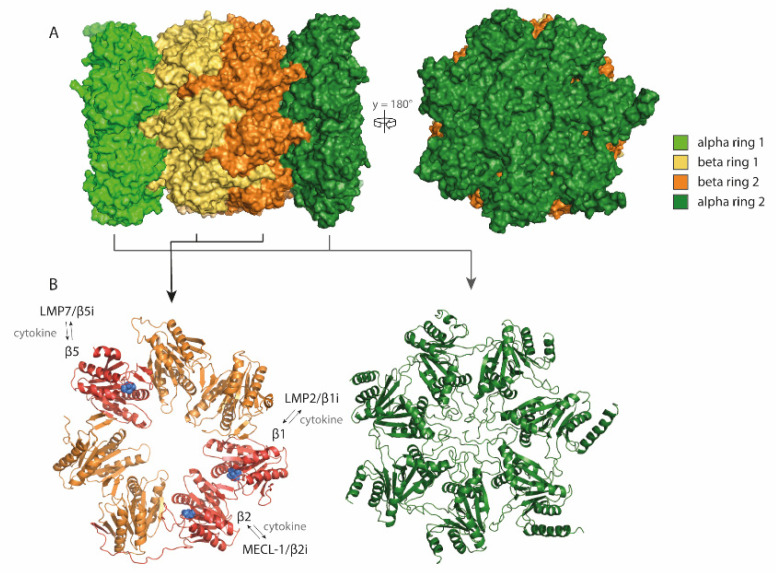
The 20S proteasome consists of two alpha and two beta rings, together forming a barrel-like particle. (**A**) Four rings of the proteasome complex stacked to form a barrel-like particle, alpha and beta rings depicted in green and yellow/orange, respectively; (**B**) Each proteasome ring consists of seven subunits: α1-7 for alpha rings and β1-7 for beta rings. Catalytic subunits are shown in red with catalytic sites indicated (blue spheres). This figure is based on the human proteasome structure: PDB file 5L4G [8].

**Figure 2:**
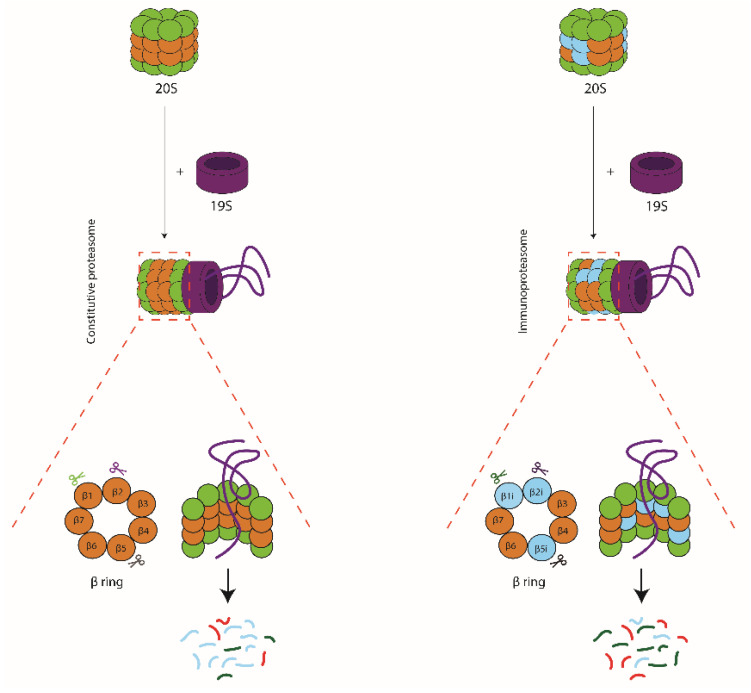
Schematic representation of 26S proteasomes, consisting of a 20S catalytic core particle attached to a 19S regulatory particle. Substrates are captured by the 19S complex, unfolded, and translocated into the 20S lumen where they are degraded by the catalytic subunits β1, β2, and β5 in constitutive proteasomes (**left hand panel**) or LMP2/β1i, MECL-1/β2i, and LMP7/β5i in immunoproteasomes (**right hand panel**). The two types of proteasomes share most cleavage sites but use these with distinct frequencies, leading to a very different representation of the single peptides within the pool of degradation products.

The ubiquitin-proteasome system is a tightly regulated proteolytic pathway responsible for degradation of proteins that localize to the cell nucleus or cytosol. In this pathway, the ubiquitination machinery targets proteins for degradation by the covalent attachment of multiple ubiquitin moieties. These are recognized by the proteasome which then unfolds and degrades the substrate (for review see [1]). Proteasomes were first identified in the eighties as multicatalytic proteinase complexes present in all eukaryotic cells [2,3,4], but in a simpler form, also exist in archaea and some eubacteria [5,6]. They appear as barrel-shaped particles, in eukaryotes composed of four stacked rings of seven subunits each, with catalytic activity restricted to three subunits in the inner two beta-rings (β1, β2 and β5) (Figure 1). These 20S catalytic core particles associate with 19S regulatory complexes to form 26S proteasomes. The 19S regulators are responsible for substrate capture, unfolding, and translocation into the 20S catalytic lumen, where the substrate is then degraded (Figure 2) [7]. Ubiquitin-dependent proteasome-mediated protein degradation plays a role in many cellular processes, including cell cycle progression, transcription factor activation, cellular stress responses, and overall protein homeostasis [6].

**Figure 3:**
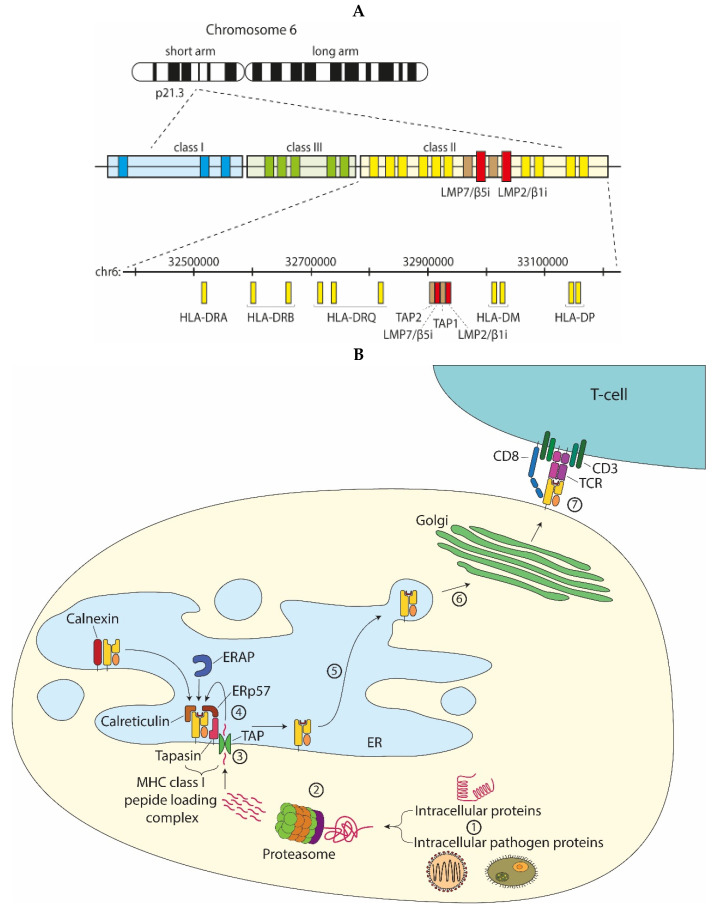
Proteasomes and MHC class I antigen processing. (**A**) The MHC class II region, in humans on chromosome 6p, encodes the two facultative proteasome subunits LMP7/β5i and LMP2/β1i, adjacent to the TAP heterodimer; (**B**) Intracellular proteins or pathogen-derived proteins (1) that are targeted for degradation are recognized by the 19S proteasome, unfolded and then degraded by the 20S proteasome (2). The proteolyzed peptides are transported from the cytosol into the ER lumen by the transporter associated with antigen processing (TAP) (3). ER-resident aminopeptidases (ERAPs) may trim the peptide N-terminus to generate peptides with an appropriate size for MHC class I binding. MHC class I chaperones stabilize the empty MHC class I molecules, assist in peptide loading, or perform quality control to select the best binding peptide (4). Upon peptide binding, the peptide-MHC class I complex dissociates from the peptide loading complex and traffics through the ER to the Golgi complex (6) and subsequently to the cell surface for presentation to cytotoxic CD8^+^ T-cells. The T-cell receptor (TCR) in complex with CD3 and CD8 on the T-cell surface binds to the peptide-MHC class I complex and upon recognition induces activation of the CD8^+^ T-cell (7).

In the early 90s, sequencing of the MHC class II region on the human chromosome 6p led to the identification of two facultative proteasome subunits/low molecular mass polypeptides (LMP) 2 and 7 (Figure 3A), interspersed between the genes thought to encode the recently identified transporter associated with MHC class I antigen processing (TAP) (Figure 3B) [9]. At that time, this discovery provided a missing link in the recently discovered MHC class I antigen processing pathway (Figure 3B), explaining where the peptides found in the MHC class I antigen-binding cleft that triggers CD8^+^ T-cell-mediated immunity, were derived from [10]. Indeed, the sequences of these two LMPs not only resembled known proteasome components; they also were inducible by IFNγ and two-dimensional gel analysis showed that complexes immunoprecipitated with anti-proteasome and anti-LMP antibodies were identical [11,12,13,14]. Additional studies convincingly showed that expression of the two LMP subunits, as well as of a third IFNγ-inducible subunit encoded outside of the MHC class II region [15,16] led to an exchange of the constitutively expressed β1, β2, and β5 proteasome catalytic sites for their inducible homologues (LMP2/β1inducible (i), multicatalytic endopeptidase complex subunit-1 (MECL-1/β2i and LMP7/β5i or the ‘immunoproteasome subunits’) in newly formed (immuno)proteasome complexes (Figure 1 and Figure 2) [17]. Tracing back in evolution, it appears that these inducible proteasome subunits first emerge in jawed vertebrates, together with MHC molecules, B- and T-cell receptors [5]. In some ectothermic, cold-blooded species, the immunoproteasome subunits are in close linkage with both TAP- and MHC class I-encoding genes and co-segregation of polymorphisms in LMP7/β5i and TAP with MHC class I polymorphisms are observed. Taken together, these data strongly suggest an important role for the immunoproteasome subunits in MHC class I antigen processing (see below).

Following the discovery of the LMP genes in the MHC class II region, different types of studies confirmed the postulated role of proteasomes in MHC class I antigen processing. Cell-membrane-permeable proteasome inhibitors were shown to inhibit antigen processing of MHC class I-presented peptides in general, and of pathogen-derived or otherwise introduced antigens in specific [18,19,20]. Furthermore, purified proteasome complexes processed antigens into MHC class I-presentable peptides [21,22]. In vitro substrate digestion studies showed that the exchange of the constitutive proteasome catalytic sites by their inducible homologues altered the peptidase activity of proteasome complexes, as well as their cleavage site preferences [22,23]. Moreover, genetically-modified mice lacking either of the two LMP subunits showed deficits in antigen processing, in CD8^+^ T-cell responses as well as T-cell development [24,25].

Following these findings, a multitude of studies since the early nineties has focused on the contribution of immunoproteasomes to antigen processing, and their impact on the immunodominance hierarchy of pathogen-specific CD8^+^ T-cell responses. These studies have revealed additional roles of immunoproteasomes in immune responses as well as novel approaches based on immunoproteasome-selective proteasome inhibitors, to treat both auto-immune diseases and cancer. Nevertheless, while the proteasome as such was found to play an essential role in the classical MHC class I antigen processing pathway [21,22,23,24], the role of the inducible subunits seemed confined to the processing and presentation of a subset of antigens [26,27]. These findings evoked the question of what precise immunological relevance the immunoproteasome subunits might have. In the following, we will review our current insights into the importance of immunoproteasome formation for the functioning of the immune system.

## 2. Role of Immunoproteasomes in MHC Class I Antigen Processing and CD8^+^ T-Cell Responses

### 2.1. Role of the Inducible Proteasome Subunits Based on Expression Patterns

Based on their location in the genome, the expectation was that the expression of the immunoproteasome subunits would be co-regulated with that of other components of the MHC class I antigen processing pathway. Northern and Western blot analyses showed that the immunoproteasome components indeed were mainly expressed in lymphoid tissues, including thymus, lymph nodes, and spleen [28], which was in agreement with the high expression levels observed in cells of the hematopoietic lineage, such as dendritic cells [29,30]. Exposure to cytokines associated with Th1-skewed immune responses, such as type I or II interferons (IFN) and TNFα, was found to (further) induce the expression levels of immunoproteasome subunits in most cells in tissue culture, along with upregulated expression of MHC class I molecules [31,32,33]. Thus, these initial analyses showed that immunoproteasome expression was mainly confined to cells of the immune system or IFN/TNFα-exposed cells.

Remarkably, a recent publication reported the generation of mutant mouse line N-ethyl-N-nitrosourea mutagenesis, carrying a missense point mutation in the gene encoding the proteasome subunit MECL-1/β2i/MECL-1 [34]. This mutation resulted in a single amino acid exchange, G170W, in MECL-1/β2i, which disrupted proteasome assembly in immune cells and led to a severe immunological phenotype and the death of predominantly B-cells. The selectivity of the consequences of this mutation to the lymphoid compartments indirectly confirms the predominant expression of immunoproteasome subunits in lymphoid tissues.

A similar conclusion can be derived from an analysis of publicly available RNAseq data sets representing 55 human tissues and cells [35]. Also, these studies showed an enrichment of the immunoproteasome subunits in lymphoid tissues. Thus, immunoproteasomes appear to play a particularly important role mainly in lymphoid tissues as well as in peripheral tissues during Th1-skewed immune responses, aimed at controlling infections by intracellular pathogens.

### 2.2. In Vitro Digestion Analyses to Reveal Immunoproteasome Function

To obtain more insight into the physiological relevance of immunoproteasomes, different groups have followed an in vitro approach in which 20S proteasome complexes were purified from cultured cells and then used to digest protein or polypeptide substrates [20,21,22,36,37,38]. Cleavage products were analyzed by LC-MS/MS analysis to identify 20S cleavage site usage and the generation of known virus- or model-antigen (ovalbumin)-derived CD8^+^ T-cell epitopes. This approach accurately reproduced in vivo epitope generation, as shown in parallel experiments in which recognition of virus-infected or antigen-transfected cells by epitope-specific CD8^+^ T-cells was tested [20,21,22,36,37,38]. Overall, most of these in vitro experiments showed that incorporation of the immunoproteasome subunits changed the frequencies of proteasome cleavage site usage, rather than introducing novel cleavage sites. For example, using cells in which expression of the three immunoproteasome subunits was controlled by a tetracycline-repressed promotor [20], it was found that cells infected with Adenovirus type 5 mutant dl7001, lacking the E3 region, processed and presented an early 1B protein-derived epitope (E1B_192–200_) more efficiently when they expressed the immunoproteasome subunits compared to when they failed to express these. In vitro digestion of a synthetic E1B polypeptide (E1B_176–215_) with purified proteasomes of the same cells cultured with or without TET demonstrated that immunoproteasomes liberated both the epitope N- and C-terminus more efficiently than constitutive proteasomes containing the β1, -2, and -5 catalytic sites.

While most studies focused on the liberation of specific model epitopes, Toes et al. [39] compared the constitutive and immunoproteasome cleavage patterns in a larger substrate, enolase-1 (436 aa). Quantitation of >120 liberated peptides by mass spectrometry and Edman sequencing showed that the two types of proteasomes displayed partially overlapping but distinct cleavage site preferences. Importantly, immunoproteasomes appeared to favor hydrophobic residues and disfavor charged residues at P1, in line with the preference of most human and mouse MHC class I alleles for peptides with hydrophobic C-termini.

To document the differences in cleavage patterns more precisely, Mishto et al. [40] performed a detailed biochemical analysis of usage 101 proteasome cleavage sites in 4 polypeptide substrates, by constitutive and immunoproteasomes of a variety of mouse and human cell lines. Likewise, Winter et al. [41] used 228 rationally designed polypeptide substrates to compare the cleavage specificities of human constitutive and immunoproteasomes. These experiments confirmed the earlier detected increased cleavage frequency by immunoproteasomes after hydrophobic and also basic residues [36,39,40,41]. However, these studies also revealed a large overlap in substrate specificity between the two types of proteasomes. In conclusion, the differences in peptide production between constitutive and immunoproteasomes mainly result from differing preferences for the usage of shared cleavage sites (Figure 2). Nevertheless, these mainly quantitative differences translate in vast differences in epitope abundance in digests, as well as in kinetics and quantities of epitope presentation by MHC class I molecules on infected cells [20,36,37,38].

### 2.3. Immune Responses in the Absence of Immunoproteasome Subunits

To determine the functional relevance of immunoproteasome expression, several groups have generated gene-deficient mice lacking one, two, or all three inducible proteasome subunits [24,25,42,43,44]. The initial studies in mice lacking LMP2/β1i [25] or LMP7/β5i [24] showed a substantial reduction of MHC class I expression in the absence of LMP7/β5i, and a reduced the presentation of specific antigens, as well as defects in T-cell development, leading to reduced numbers of antigen-specific CD8^+^ T-cell precursors, in LMP2/β1i-deficient mice. Subsequent studies further refined these initial observations: in the absence of LMP7/β5i, a significant reduction in MHC class I levels, to approximately 50 to 60% of the levels in wild type (WT) mice, was observed [43,44,45,46]. Mice lacking LMP2/β1i or MECL-1/β2i exhibited defects in the CD8^+^ T-cell repertoire [42,47], and diminished numbers of CD8^+^ relative to CD4^+^ T-cells in the periphery [43,45]. Upon infection with pathogens, such as influenza virus, Lymphocytic Choriomeningitis Virus (LCMV), and *Listeria monocytogenes*, pronounced differences in the immunodominance hierarchies of induced responses were observed in all single or double (LMP7/β5i and LMP2/β1i or MECL-1/β2i) gene-deficient strains [37,47,48,49]. In all studies, drastically reduced responses were detected to several epitopes that triggered immunodominant responses in WT mice, due to their reduced presentation by MHC class I molecules [47,48,49], or, alternatively, to changes in T-cell repertoire [42,47]. In a few studies, increased responses to otherwise subdominant epitopes were observed, probably resulting from enhanced epitope generation in the absence of a preferred immunoproteasome’ cleavage site [37,49], or increased possibilities for T-cell expansion in the absence of a dominating response [50,51,52,53]. Importantly, in mice lacking all three immunoproteasome subunits, generated by Kincaid et al. [44], CD8^+^ T-cell responses to most epitopes studied were reduced, including epitopes for which no prior defects were detected in single gene-deficient mice. Mass spectrometry analysis of peptides eluted from MHC class I molecules of mouse splenocytes showed a discordance of as much as 50% between MHC class I–presented peptides in WT and these triple immunoproteasome subunit gene-deficient mice. In line with this difference in peptide repertoire, CD8^+^ T-cells in triple gene-deficient mice rejected WT splenocytes, as observed also in earlier studies [24,39]. Taken together, these data indicate that the replacement of the constitutive proteasome subunits for their inducible homologues in infected and lymphoid tissues, is required for efficient antigen presentation, as well as T-cell repertoire development. Thereby, immunoproteasome formation determines the specificity and magnitude of CD8^+^ T-cell responses triggered following infection. Of note, this effect is limited to the CD8^+^ T-cell compartment; CD4^+^ T-cell responses were found to be unaffected by any changes in proteasome subunit composition [44,46].

### 2.4. Role of Immunoproteasomes in Immune Protection

The studies reviewed above illustrate the changing views on immunoproteasome function: from ‘possibly’ essential’ for antigen processing after the discovery of the LMPs, to ‘perhaps not so important,’ and back to ‘very significant’ based on the newer publications using triple gene-deficient mice. In addition to above-described publication, numerous other manuscripts have demonstrated additional functions for immunoproteasomes in protein homeostasis, cytokine secretion, and T-cell differentiation [54,55,56,57,58,59,60]. Despite all these different roles attributed to immunoproteasomes, their impact on one of the most important functions of the immune system, the expulsion of pathogens, has remained understudied. Although the antigen specificity of CD8^+^ T-cell responses mounted in mice lacking one or two immunoproteasome subunits differs, these mice are capable of clearing viral pathogens [61]. LMP7/β5i deficiency, on the other hand, was found to increase susceptibility of mice to *Toxoplasma gondii*, an intracellular parasite [62]. More recently, the triple immunoproteasome subunit gene-deficient mice of Kincaid et al. were tested for their ability to eliminate *Trypanosoma cruzi*, a human protozoan parasite controlled by CD8^+^ T-cells [63]. Compared to WT controls, infected triple gene-deficient bone marrow-derived dendritic cells (BM-DC) showed a diminished MHC class I presentation of *T. cruzi* antigens. CD8^+^ T-cells in triple gene-deficient mice poorly responded to infection and failed to control parasite loads, and mice finally succumbed to infection [63]. In addition, Guimaraes et al. [64] demonstrated a delayed clearance, diminished antigen presentation and diminished CD8^+^ T-cell responses in triple gene-deficient mice infected with the intracellular bacterium *Brucella abortus*. Taken together, these studies in different mouse models indicate that immunoproteasome formation may play an important role in pathogen resistance.

### 2.5. Exploiting Immunoproteasomes to Control Infections

Recently, significant progress was made towards the development of vaccines triggering protective immunity to intracellular pathogens [65,66,67,68,69,70]. For example, current COVID-19 vaccines express the viral spike protein to induce both neutralizing, humoral responses and CD8^+^ T-cell-mediated immunity [67,68,69,70,71]. The elicited humoral responses, as monitored in standard assays, confer sterilizing immunity against the original SARS-CoV-2 virus, but appear to protect less well against newer virus variants that have accumulated a variety of mutations in their spike sequences [71]. Contrary to neutralizing antibodies, the fine specificity of vaccine-induced CD8^+^ T-cells due to HLA polymorphism differs between individuals, which impedes viral evasion. Thus, although their role is presently unclear, vaccine-induced CD8^+^ T-cell responses may significantly contribute to protection against such virus variants.

Due to lacking knowledge on the nature of effective CD8^+^ T-cell responses, the optimal vaccine capable of eliciting an effective CD8^+^ T-cell response has yet to be designed. Nevertheless, different studies have taught us some guiding principles. In the first place, the studies reviewed above suggest that (i) infected cells and immune cells including professional APC express high levels of immunoproteasomes, and (ii) immunoproteasome formation increases the processing efficiency of many known immunodominant CD8^+^ T-cell epitopes. Possible effects of such increased processing efficiency were illustrated by Deol et al. [48], who constructed a recombinant *Listeria monocytogenes* strain (rLM-E1) secreting a hybrid antigen. This antigen contains the immunoproteasome-generated Ad5 E1B epitope in context of its natural flanking sequences. Using E1B-specific CD8^+^ T-cells as a read out, it was shown that following infection with rLM-E1, BM-DC lacking LMP2/β1i and LMP7/β5i processed and presented the E1B epitope substantially slower than WT BM-DC. In line with these findings, infected LMP2/β1i and LMP7/β5i gene-deficient mice failed to mount CD8^+^ T-cell responses to this epitope, which was dominant in WT mice. In contrast, immunization with DC pulsed with synthetic E1B triggered E1B-specific responses in both WT and LMP2/β1i and LMP7/β5i-deficient mice; these T-cells formed memory and expanded upon later challenge with rLM-E1, in both mouse strains. In addition, splenic APC of T-cell-depleted LMP2/β1i and LMP7/β5i-deficient mice, infected for 36 h with rLM-E1, triggered E1B-specific CD8^+^ T-cell responses upon transfer into WT, as well as LMP2/β1i and LMP7/β5i-deficient mice. Thus, given sufficient time, LMP2/β1i and LMP7/β5i-deficient splenic APC processed rLM-E1-derived E1B in sufficient quantities to trigger specific CD8^+^ T-cells, but LMP2/β1i and LMP7/β5i-deficient mice failed to respond to this epitope (while responding to control epitopes) following primary infection. Taken together, these data strongly suggest that epitopes need to reach a certain threshold level at the pAPC surface early in infection, in order to successfully prime CD8^+^ T-cell responses. Similar observations were made by Zanker et al. [49] but in secondary infection, showing that immunodominance hierarchies to 9 Influenza epitopes in WT and different immunoproteasome subunit-deficient mice correlated with the kinetics of antigen presentation of each of these epitopes. Furthermore, Wu et al. [72] explored the principles behind immunodominance hierarchies quantified MHC class I presentation of 21 influenza-virus-derived peptides by mass spectrometry. They found that all epitopes that elicited readily detectable CD8^+^ T-cell responses in primary infection were highly abundant in either infected (six epitopes) or cross-presenting (one epitope) cells. A possible explanation for these observations is offered by a variety of studies demonstrating competition between CD8^+^ T-cells for priming by antigen-presenting cells [51,73,74], jeopardizing CD8^+^ T-cells specific for epitopes that are produced with relatively slow kinetics. Taken together, although many other factors such as MHC-peptide binding affinity, ‘immunodomination’ of specific T-cells over others [53] and co-expressed HLA alleles [75] may contribute to epitope dominance, the above data strongly suggest that a new generation of CD8^+^ T-cell eliciting vaccines should take antigen processing kinetics and early epitope abundance into account. Given the rapid advances in the biomedical field and novel opportunities offered by computational biology, ensuring efficient epitope presentation from vaccine vectors in the different target populations should be feasible in the near future.

## 3. Proteasome Subunit Composition and T-Cell Selection

During their development in the thymus, immature thymocytes rearrange their T-cell receptor gene segments to form a functional TCR. To select a broad TCR repertoire specific for MHC-presented peptides of foreign antigens, thymocytes first are positively selected for recognition of self-MHC in the thymic cortex and then negatively selected in the medulla, to eliminate autoreactive T-cells. Thymocyte fate during these selective processes is determined by the strength of signals received through the TCR: intermediate affinity interactions with MHC-self peptide complexes (MHC/pep) on cortical thymic epithelial cells (cTECs) stimulate thymocyte survival, while high-affinity interactions with MHC/pep on medullary (m)TECs and DC in the medulla lead to apoptosis. Consequently, as producers of the MHC class I-presented peptides, proteasomes play an important role in the selection of the CD8^+^ TCR repertoire.

Remarkably, approximately two decades after the discovery of the immunoproteasome subunits, Murata et al. [76] found a third homologue of β5, β5t, that coevolved with the immunoproteasome subunits [5]. The gene encoding β5t is located adjacent to the β5 encoding gene and selectively expressed in cTECs. β5t in cTECs joins with LMP2/β1i and MECL-1/β2i to form ‘thymoproteasomes’ in these cells, producing the ligands that determine positive selection [76]. DC and mTECs in the medulla, on the other hand, lack β5t but express LMP2/β1i, MECL-1/β2i, and LMP7/β5i, leading to immunoproteasome formation in these cells.

Given the different functions of cTECs and mTECs/DC in TCR repertoire selection, it is tempting to speculate that the set of peptides produced by thymoproteasomes fundamentally differs from the immunoproteasome-produced one that is presented in the thymic medulla and during immune responses in the periphery. In support of this, Murata et al. [76] showed that incorporation of β5t reduced the capacity of proteasomes to cleave after hydrophobic residues. In contrast, this activity is enhanced by the incorporation of LMP7/β5i [22,23,39,77], to support the generation of high-affinity MHC class I ligands [24]. In line with the altered catalytic properties of β5t, thymoproteasomes were found to exhibit unique cleavage preferences in polypeptide substrates leading to the liberation of a distinct set of peptides, which only partially overlapped with the peptides produced by immunoproteasomes [78]. In line with a strong influence of thymoproteasomes on positive selection, mice lacking β5t showed a significant reduction in percentages and numbers of CD8^+^ but not CD4^+^ T-cells in the thymus and periphery [76]. Moreover, CD8^+^ T-cells in β5t-deficient mice exhibit an altered TCR repertoire, poorly respond to allogeneic stimuli and these mice show an enhanced susceptibility to influenza virus infection compared to heterozygotic mice expressing a single copy of the β5t gene [79]. To further examine the effects of altered peptide display on cTECs on T-cell selection, Xing et al. [80] generated a β5t-deficient LMP7/β5i knock-in mouse strain and then crossed these mice with LMP7/β5i-deficient mice. The generated mice express LMP7/β5i from the β5t locus but not elsewhere, leading to the formation of immunoproteasomes exclusively in cTECs. Remarkably, despite the immunoproteasome-mediated production of a distinct set of MHC class I ligands in cTECs, these mice failed to select a large repertoire of CD8^+^ T-cells, suggesting that thymoproteasome-generated peptides display specific intrinsic properties supporting positive selection. Moreover, Kincaid et al. [81] generated mice lacking β5t, as well as all three immunoproteasome subunits. These mice that express constitutive proteasomes in all cells including cTECs and mTECs showed a severely impaired CD8^+^ but not CD4^+^ thymocyte development, due to defects at the level of both positive and negative selection. Taken together, these data strongly suggest that the unique properties of thymoproteasome-generated peptides optimize positive selection, while a switch in MHC class I-presented peptides between cTECs responsible for positive selection, and mTECs and DC responsible for negative selection, is essential for overall T-cell development [80,81]. Nevertheless, the exact mechanism by which β5t generated peptides influence CD8^+^ T-cell selection remains unresolved.

## 4. Role of Immunoproteasomes in Auto-Immune Responses

### 4.1. CD8 T Cell-Mediated Early Stage, Multi-Tissue Autoimmune Disease in Immunoproteasome Subunit-Deficient Mice

In line with their role in T-cell selection (see above), the first analyses of both LMP2/β1i and MECL-1/β2i gene-deficient mice readily revealed that their T-cell repertoire differed from that in WT mice, leading to diminished CD8^+^ T-cell responses to specific viral epitopes upon infection [42,47]. Remarkably, in an analysis of MECL-1/β2i and LMP7/β5i double gene-deficient mice, Zaiss et al. [82] noticed the development of multiple autoimmune syndromes including dermatitis, diabetes insipidus, and latent insulin-dependent diabetes mellitus (IDDM) following full-body irradiation and bone marrow transfer. Disease symptoms were caused by CD8^+^ T-cells and developed following engraftment with either MECL-1/β2i and LMP7/β5i deficient or WT bone marrow in gene-deficient but not in WT mice. In diseased mice, CD8^+^ T-cells specific for four IDDM-associated epitopes out of four tested, one derived from pro-insulin and three from islet-specific glucose-6-phosphatase catalytic subunit-related protein (IGRP), were detected. Moreover, CD8^+^ T-cells of diseased mice transferred the disease phenotype onto MECL-1/β2i and LMP7/β5i -deficient but barely to MECL-1/β2i and LMP7/β5i-sufficient recombination-activating gene 1 (RAG1)-deficient mice. Taken together, these data showed a remarkable co-segregation of disease with the absence of immunoproteasomes in the inflamed tissue, and not with a mismatch between the recipient and BM donor. Given the variety of epitopes recognized, it was speculated that MECL-1/β2i and LMP7/β5i-deficient tissue may be more prone to stress-induced cell death, leading to the priming of autoreactive CD8^+^ T-cells. Such an enhanced susceptibility to stress-induced cell death and autoimmune disease was shown for example by Seifert et al. [55], in a study aimed to examine the consequences of immunoproteasome deficiency for overall cellular protein homeostasis. An alternative or complementary explanation however may lay in the properties of the epitopes recognized in autoimmune diseases. These often bind their presenting MHC class I molecule with relatively low affinity [83], as shown also for one of the IGRP epitopes, newly identified in Zaiss et al. [82]. Such epitopes may be more efficiently presented in inflamed tissues under conditions of severe ligand shortage, in the absence of LMP7/β5i expression. Altered T-cell selection in the MECL-1/β2i and LMP7/β5i-deficient thymus remains a third although a less likely possibility, since three of the CD8^+^ T-cell epitopes studied had been identified in different immunoproteasome sufficient mouse models [83]. In conclusion, proteasome subunit composition may affect both T-cell selection (other CD8^+^ T-cell specificities) and the display of peptide ligands in inflamed tissues, and in this way contribute to autoimmune reactions. Such enhanced epitope display may result from enhanced MHC class I-presentation of low(er) affinity binders (a general effect) but may also result from efficient processing of specific epitopes by immunoproteasomes or mixed proteasomes in the attacked, inflamed tissue [58,84].

### 4.2. Genetic Linkage of Immunoproteasome Subunits with Different Autoinflammatory and Autoimmune Diseases

A variety of genomic studies comparing data of patients and healthy family members demonstrated an association between polymorphisms in human proteasome genes and specific diseases, including autoinflammatory and autoimmune diseases [85]. For instance, in 2010 Torello et al. [86] described an inflammatory syndrome characterized by chronic atypical neutrophilic dermatosis with lipodystrophy and elevated temperature (CANDLE). Remarkably, this disease appeared to fall into a spectrum of proteasome-associated autoinflammatory syndromes (PRAAS), associated with specific mutations in the LMP7/β5i (PSMB8) gene sequences or other proteasome genes that reduce proteasome function [87,88,89,90,91]. As also observed in murine inflammation models by Seifert et al. [55], impaired immunoproteasome function in cells and inflamed tissue of PRAAS patients leads to an accumulation of ubiquitinated and oxidized proteins [88,91], which is linked to a chronically enhanced type I interferon production and expression of interferon-induced chemo- and cytokines [91]. To explain the link between immunoproteasome dysfunction and interferonopathy, Ebstein et al. [92] suggested that defective protein degradation causing the formation of proteotoxic aggregates may activate the unfolded protein response. This in turn may induce type I interferon production and initiate autoinflammatory disease in PRAAS patients.

Focusing on type 1 diabetes (T1D), Zaiss et al. [82] analyzed the T1D Genetics Consortium dataset which contains data on 1557 SNPs across the MHC region for 2321 T1D families. This study detected two linked SNPs in PSMB8 that, conditional on the main genetic HLA determinant DRB1-DQA1-DQB1, were significantly associated with T1D development. Linkage disequilibrium between these SNPs and T1D was especially strong in the context of the T1D predisposing HLA B8 and B18 alleles (OR > 3), implicating the SNP-marked PSMB8 allelic form as a risk factor for T1D development. Also, Xu et al. [93] analyzing metadata, derived from literature searches, of Asian, African, and Caucasian populations detected two allelic forms of LMP2/β1i and LMP7/β5i that were significantly associated with T1D, conferring susceptibility and protection, respectively. In line with these findings, several recent transcriptome and interactome studies implicated the proteasome genes in the biological processes leading to T1D, as well as multiple sclerosis [94,95]. In a smaller study in Latvians [96], genetic variations in three non-catalytic 26S proteasome subunits were identified as risk factors in T1D. Thus, in line with results in experimental models, also the analyses of immunogenetics data of patient cohorts implicate the (immuno)proteasome in specific autoimmune diseases in which CD8^+^ T-cells contribute to the observed immunopathogenesis.

### 4.3. Targeting Immunoproteasomes to Dampen Auto-Immune and Inflammatory Disease

As apparent from the studies discussed above, immunoproteasomes play a more central role during immune responses than just as providers of MHC class I ligands [97,98]. In mouse models, in addition to a possible (but debated) role in the maintenance of protein homeostasis in inflamed tissues [55,99,100,101], several studies reported a dysregulated signaling through the NFkB pathway in the absence of specific immunoproteasome subunits [54,60], reduced cytokine production by T-cells and macrophages, and reduced skewing of Th cells towards the Th1 and Th17 phenotype [57,58,60]. Therefore, there are multiple levels at which immunoproteasomes may influence immunopathology in autoimmune or inflammatory diseases. This complexity at times may perturb interpretation of obtained results. For example, mice lacking LMP7/β5i expression were found to be less susceptible to the development of dextran sulfate sodium-induced colitis, a disorder in which monocyte-derived macrophages play an important role [57,60]. On the other hand, in a CD4^+^ T-cell transfer model, LMP2/β1i + MECL-1/β2i -deficient T-cells produced more IL17 and were more potent than WT T-cells in inducing colitis when transferred into RAG1^-/-^ mice [102]. Thus, the effects of proteasome subunit composition on disease susceptibility may depend on the precise immune effector mechanisms responsible for disease development.

Notwithstanding its differing roles in different disease models, the immunoproteasome due to its selective expression in immune cells and inflamed tissues provides an excellent target for interference with autoimmune and inflammatory diseases [98]. In an early study, Muchamuel et al. [58], showed that inhibition of immunoproteasomes using an LMP7/β5i selective drug diminished the production of pro-inflammatory cytokines such as IL23, TNFα, and IL6 by LPS-stimulated monocytes, and reverted disease progression of both collagen-induced and collagen antibody-induced arthritis in mouse models. Since then, numerous immunoproteasome-selective inhibitors have been developed and tested in a large variety of autoimmune as well as other (inflammatory) disorders, in animal models and in clinical settings (for review see [103,104,105]). Noteworthy, despite initial successes with LMP7/β5i -selective inhibitors, different studies showed that treatments were most effective when both LMP7/β5i and either MECL-1/β2i or LMP2/β1i activity were blocked [104,106]. In agreement with their specificity for immunoproteasomes, this newer generation of inhibitors seems less toxic than an earlier generation of more general proteasome inhibitors, which includes the FDA- and EMA-approved approved drugs Bortezomib and Carfilzomib, applied for the treatment of hematological malignancies [106]. None of the more selective immunoproteasome inhibitors has currently advanced to clinical application, but in their current or more optimized form may become available for the treatment of a wide range of inflammatory diseases in the near future.

## 5. Concluding Remarks

The discovery of the genes encoding two IFNγ-inducible proteasome subunits in the MHC class II region, back in the 90s of the last century, suddenly placed the proteasome in the middle of the then just uncovered MHC class I antigen processing pathway. Since then, many studies have been devoted to these and a third IFNγ-inducible proteasome subunit, to elucidate their contribution to the functioning of the immune system. It has become apparent that cells in infected or inflamed as well as lymphoid tissues express the inducible proteasome subunits LMP2/β1i, MECL-1/β2i, and LMP7/β5i, which are preferentially incorporated into newly assembled proteasomes. The altered catalytic properties of the so-formed immunoproteasomes were found to play a critical role in the processing and presentation of MHC class I ligands and to determine the fine specificity of CD8^+^ T-cell responses to intracellular pathogens. However, proteasome subunit composition was also shown to play a role in the selection of T cells during their development in the thymus. Here, the expression of the thymosubunit β5t in cTECs was found to optimize the positive selection of T cells, while immunoproteasome expression in mTECs and DC supports negative selection to warrant the clonal deletion of auto-reactive T cells. Thus, immunoproteasomes, as the major proteolytic forces in cells, mediate their function at different levels, including T-cell selection and epitope production, and so shape the protective CD8^+^ T cell responses to intracellular pathogens.

Given their role in CD8^+^ T cell immunity, one would speculate that defects in immunoproteasome function could contribute to autoimmune diseases in which CD8^+^ T cells play a role. Both studies in mouse models and meta-analyses of human genetic data indeed have linked immunoproteasome subunit deficiency (in mice) and specific polymorphisms in the immunoproteasome subunit genes LMP2/β1i and LMP7/β5i (in humans) to different, in part CD8^+^ T-cell-mediated autoimmune syndromes such as T1D. However, other more recent human genetic studies also revealed a group of autoinflammatory syndromes with a strong type I IFN signature, that was caused by loss-of-function mutations in several proteasome genes including LMP7/β5i. Thus, the effects of immunoproteasomes reach far beyond their role in antigen processing. This is apparent also from different murine autoimmune and inflammatory disease models where immunoproteasome subunit deficiency or inhibition was shown to modulate the production of (pro-)inflammatory cytokines and Th cell differentiation.

We conclude that since their initial discovery, a profound understanding of the characteristics and functions of the immunoproteasome subunits has been gained. Now, this gained knowledge is translated into novel strategies for clinical immune intervention. Catalytic site-selective inhibitors are already tested for their ability to ameliorate hematological, inflammatory, and autoimmune disorders, and with the rapid advances in personalized medicine, immunoproteasome cleavage site preferences may be further exploited to develop effective vaccines against ‘difficult’ intracellular pathogens or cancers for specific patient populations.

## Data Availability

Not applicable.
